# Breathing and burning: metabolic control of alveolar macrophages by intracellular bacterial pathogens

**DOI:** 10.3389/fphys.2026.1895061

**Published:** 2026-09-03

**Authors:** Het Adhvaryu, Daniel E. Voth

**Affiliations:** Department of Microbiology and Immunology, University of Arkansas for Medical Sciences, Little Rock, AR, United States

**Keywords:** alveolar macrophages, immunometabolism, infection, intracellular pathogen, pulmonary, vacuole

## Abstract

Alveolar macrophages (AMs) are the lung’s phagocytic immune sentinels, programmed to maintain homeostasis while defending against inhaled pathogens. AM metabolism is shaped by both developmental origin and the alveolar microenvironment. Embryonically-derived AMs populate the lungs early in life and develop into long-lived, self-renewing cells displaying lipid-centered, oxidative metabolism that restrains inflammation and preserves alveolar structure. When infection or injury disrupts homeostasis, circulating monocytes are recruited to the airspace and differentiate into monocyte-derived AMs, which adopt a glycolytic, short-lived, pro-inflammatory phenotype. Thus, differences in origin and transcriptional programming create two metabolically distinct AM populations with divergent roles in lung immunity. The intracellular bacterial pathogens *Mycobacterium tuberculosis, Coxiella burnetii*, *Legionella pneumophila*, and *Francisella tularensis* exploit these metabolic programs to facilitate intracellular growth and disease progression. By modulating glycolysis, remodeling mitochondria, manipulating lipid handling, or redirecting host metabolites, these pathogens evade immune insults and create growth niches. In this review, we discuss mechanisms by which AM metabolism shapes the tissue environment and pulmonary immunity, and we showcase intracellular pathogens to understand pro-bacterial reprogramming of AM metabolism.

## Introduction

With every breath, the human lung is exposed to an influx of airborne pathogens, necessitating a delicate balance between immune vigilance and prevention of excess inflammation that unnecessarily damages healthy cells and tissues. Resident alveolar macrophages (AMs) are the forefront immune sentinels of the lung that continuously patrol pulmonary alveolar spaces to clear debris and neutralize inhaled microbes (Neupane et al., 2020). AMs have the challenging task of mounting an effective immune response while preserving lung homeostasis (Hussell and Bell, 2014). To perform these dual functions, AMs display dynamic metabolic activity to respond with appropriate immune activation. AMs predominantly express oxidative and lipid-driven metabolism, shaped by the oxygen-rich and surfactant-laden environment of alveoli that fuels AM-specific functions (Wculek et al., 2022). At homeostasis, AMs preferentially rely on oxidative metabolism in the presence of oxygen. During inflammation or injury, recruited and activated macrophages increasingly engage aerobic glycolysis, defined as glycolytic ATP production, despite the presence of oxygen, whereas resident AMs often retain metabolically distinct programs shaped by the pulmonary microenvironment (Woods et al., 2020; Wculek et al., 2022).

AMs originate from either embryonic precursors or circulating monocytes, and either type differentiates into AMs with distinct functions and metabolic profiles. During embryonic development, fetal monocytes seed the alveolar space and differentiate into long-lived, self-renewing AMs (Guilliams et al., 2013). Embryonically-derived AMs regulate lung homeostasis by alveolar patrolling, phagocytosis, and immune tolerance (Dong and Wright, 1998; Dong et al., 1998; Westphalen et al., 2014; Neupane et al., 2020). When homeostasis is disturbed by inflammation or infection, recruited monocytes differentiate into monocyte-derived AMs (MDAMs). Unlike embryonic-AMs, MDAMs are short-lived, transcriptionally distinct, inflammatory cells with increased glycolytic programming, impaired lipid handling, and enhanced antimicrobial functions (Misharin et al., 2017; Mould et al., 2019; T'Jonck and Bain, 2023). Embryonic AM differentiation is guided by local signals including GM-CSF and TGF-β, with downstream activation of transcription factors such as PPAR-γ that drive oxidative metabolism and anti-inflammatory programming (Guilliams et al., 2013; Yu et al., 2017). Pro-inflammatory factors, such as IL-1β and type I interferons, drive MDAM metabolism to a pro-inflammatory glycolytic state controlled by regulators such as HIF-1α (T'Jonck and Bain, 2023; Liu et al., 2025). Differences in origin and transcriptional wiring fundamentally influence AM metabolic responses to a diverse array of pathogens, forging AMs as the cellular protectors of the lung.

Dynamic metabolic activity allows AMs to mount a range of antimicrobial responses against invading pathogens. AM metabolic reprogramming upon cytokine exposure, epithelial cell crosstalk, or pattern-associated molecular signals, support pro-inflammatory functions. Activation of AMs includes production of TNF-α, IL-1β, and reactive oxygen and nitrogen species, all of which require substantial metabolic input regulated by oxidative phosphorylation (OXPHOS), glycolytic transitions, and mitochondrial remodeling (Svedberg et al., 2019; Ogger and Byrne, 2021; Cox et al., 2024). Manipulation of these metabolic events allows respiratory pathogens to evade immune recognition, modulate key signaling pathways, and redirect cellular nutrients for their own replication, making AM metabolism an attractive target for intracellular bacterial survival and persistence. Indeed, alteration of AM bioenergetics can direct pathogen clearance or persistence, defining infection in the lung environment. Intracellular pathogens must establish a growth niche within host cells, making hijacking host metabolism essential for intracellular replication.

The pulmonary intracellular bacterial pathogens *Mycobacterium tuberculosis*, *Coxiella burnetii*, *Legionella pneumophila*, and *Francisella tularensis* target metabolic processes within AMs to support their growth in human lungs. These organisms create a favorable intracellular niche by altering mitochondrial dynamics, oxidative metabolism, glycolytic switching, and/or metabolite sequestration, allowing evasion of antimicrobial defenses. Briefly, *F. tularensis* and *M. tuberculosis* suppress glycolytic activation, delaying inflammatory signaling and cell death to allow bacterial growth. *L. pneumophila* remodels mitochondria to redirect energy production toward glycolysis to facilitate bacterial infection, while *C. burnetii* adopts a more quiescent strategy, preserving OXPHOS and inhibiting apoptosis to maintain a pro-bacterial niche. Collectively, these pulmonary pathogens showcase a common reliance on host metabolism as a strategy to manipulate AM function and sustain intracellular growth.

In this review, we examine AM metabolism at the intersection of pulmonary immunity and intracellular bacterial pathogenesis. First, we explore how AM origin and the pulmonary environment shape metabolism that influences AM function to maintain homeostasis or initiate immune defense. Second, we highlight strategies used by four intracellular bacterial pathogens that each reprogram AM metabolism to establish a replication niche. These pathogens highlight an emerging field and show that behind each breath lies a dynamic metabolic battleground, one that may hold the key to defending the lungs from within.

## Alveolar macrophage metabolism during homeostasis and activation

**Figure 1:**
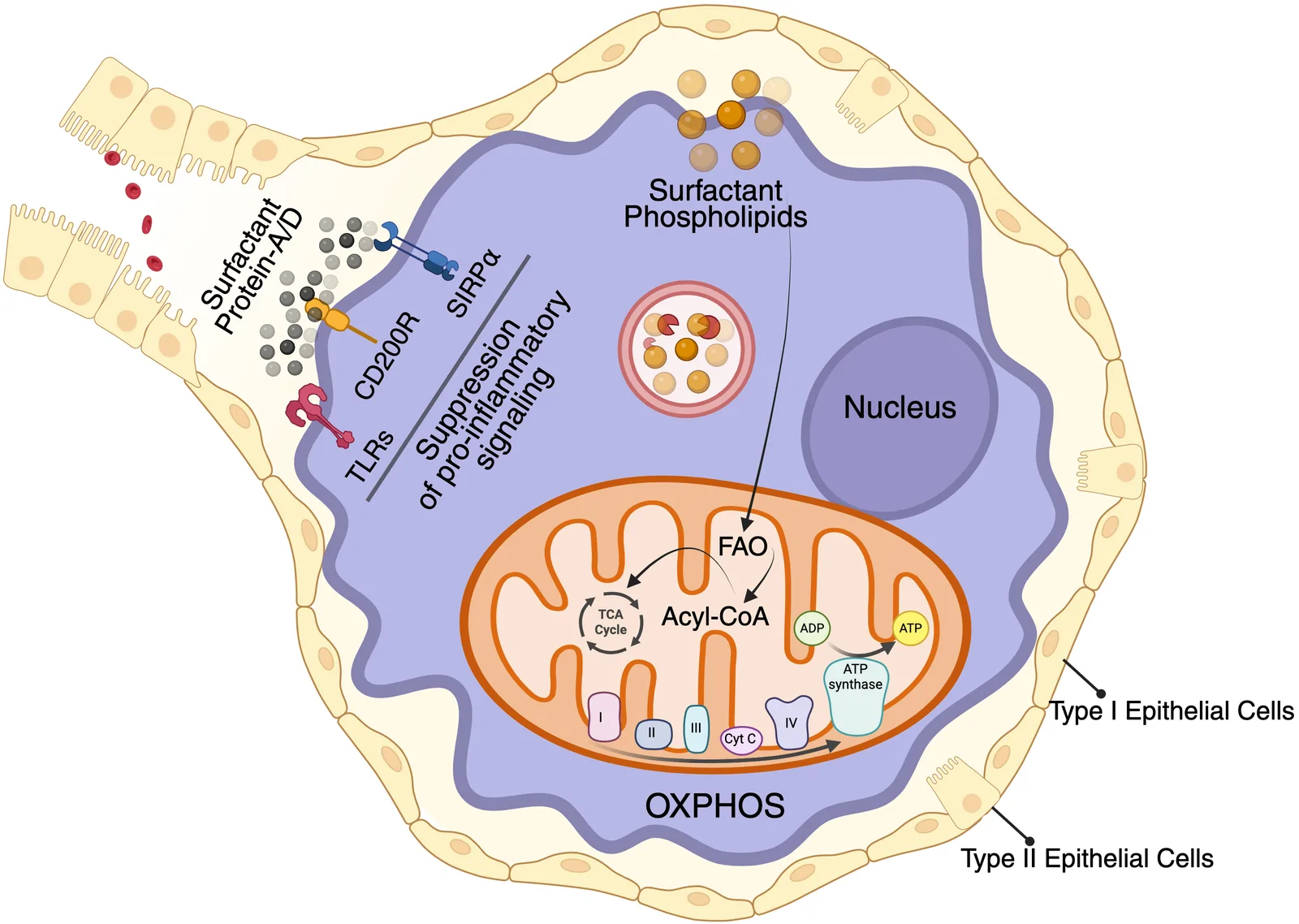
Homeostatic characteristics of AMs. AMs maintain pulmonary homeostasis through continuous surveillance of the alveolar space to clear debris and pathogens. AM function is primarily supported by OXPHOS, FAO, lipid metabolism, and cholesterol metabolism. Environmental surfactant proteins additionally engage immune receptors on AMs to promote an immunosuppressive pulmonary state.

AMs are long-lived resident phagocytes that maintain alveolar homeostasis while patrolling the lung for inhaled matter, including pathogenic microbes. At steady state, embryonically-derived AMs display a quiescent state supported by mitochondrial respiration and lipid use in the oxygen- and surfactant-rich alveolar space (Schneider et al., 2014; Yu et al., 2017). GM-CSF produced by alveolar type II epithelial cells and TGF-β made by mesenchymal cells trigger AM differentiation and long-term survival, activating PPAR-γ to promote fatty acid uptake, mitochondrial biogenesis, and OXPHOS while suppressing glycolytic and inflammatory programs (Guilliams et al., 2013; Schneider et al., 2014; Yu et al., 2017; McCubbrey et al., 2018; Ogger and Byrne, 2021). Compared to other macrophages, at homeostasis, AMs display higher basal and maximal OXPHOS with substantial spare respiratory capacity, while channeling surfactant-derived fatty acids through β-oxidation into the tricarboxylic acid (TCA) cycle, ultimately supporting continuous surfactant catabolism, cholesterol processing, and immune quiescence to maintain pulmonary homeostasis (Schneider et al., 2014; Huang et al., 2018; Wculek et al., 2022) (**Figure 1**).

AMs are continuously shaped by pulmonary signals, and embryonically-derived AMs are self-renewing and developmentally distinct from interstitial and MDAMs (Guilliams et al., 2013; Schneider et al., 2014), although circulating monocytes can contribute to the AM population following disruption of the alveolar space (Byrne et al., 2020). This GM-CSF/TGF-β-driven program is further reinforced by the pulmonary microenvironment, establishing AM-specific enhancer landscapes that sustain lipid-centered oxidative gene expression distinct from other tissue macrophages (Lavin et al., 2014; Mould et al., 2019). Specifically, GM-CSF activates PPAR-γ to promote fatty acid uptake, mitochondrial biogenesis, and oxidative phosphorylation, while TGF-β suppresses glycolytic and inflammatory programs (Schneider et al., 2014; Yu et al., 2017; McCubbrey et al., 2018). Disruption of fatty acid oxidation (FAO), OXPHOS, and cholesterol metabolism impairs surfactant catabolism resulting in accumulation of surfactant lipids and directly linking AM metabolism to tissue homeostasis (Baker et al., 2010; Schneider et al., 2014).

Inflammatory stress disturbs homeostasis and remodels AM metabolism. During infection or injury, chemokines recruit monocytes from the bloodstream that differentiate into AMs in the alveolar space (Misharin et al., 2017; Bleriot et al., 2020). MDAMs engage glycolytic metabolism associated with HIF-1α stabilization and increased glycolytic enzyme expression. This glycolytic program supports effector functions, such as inflammatory cytokine production and ROS and NO generation during acute lung injury and inflammation (Woods et al., 2022; Woods and Mutlu, 2025). Inflammatory cytokine production by these cells is closely linked to glycolytic engagement, coupling metabolic state to effector function. Recruited MDAMs have impaired lipid handling and altered mitochondrial function compared to embryonic AMs (Wculek et al., 2023), and they are transcriptionally distinct and less durable, often retaining a glycolytic phenotype post-inflammation (Misharin et al., 2017; T'Jonck and Bain, 2023). These differences highlight how AM origin and environmental cues drive divergent metabolic programs with disparate consequences for lung health and provide a framework for understanding dysregulated macrophage metabolism during pulmonary infection.

**Figure 2:**
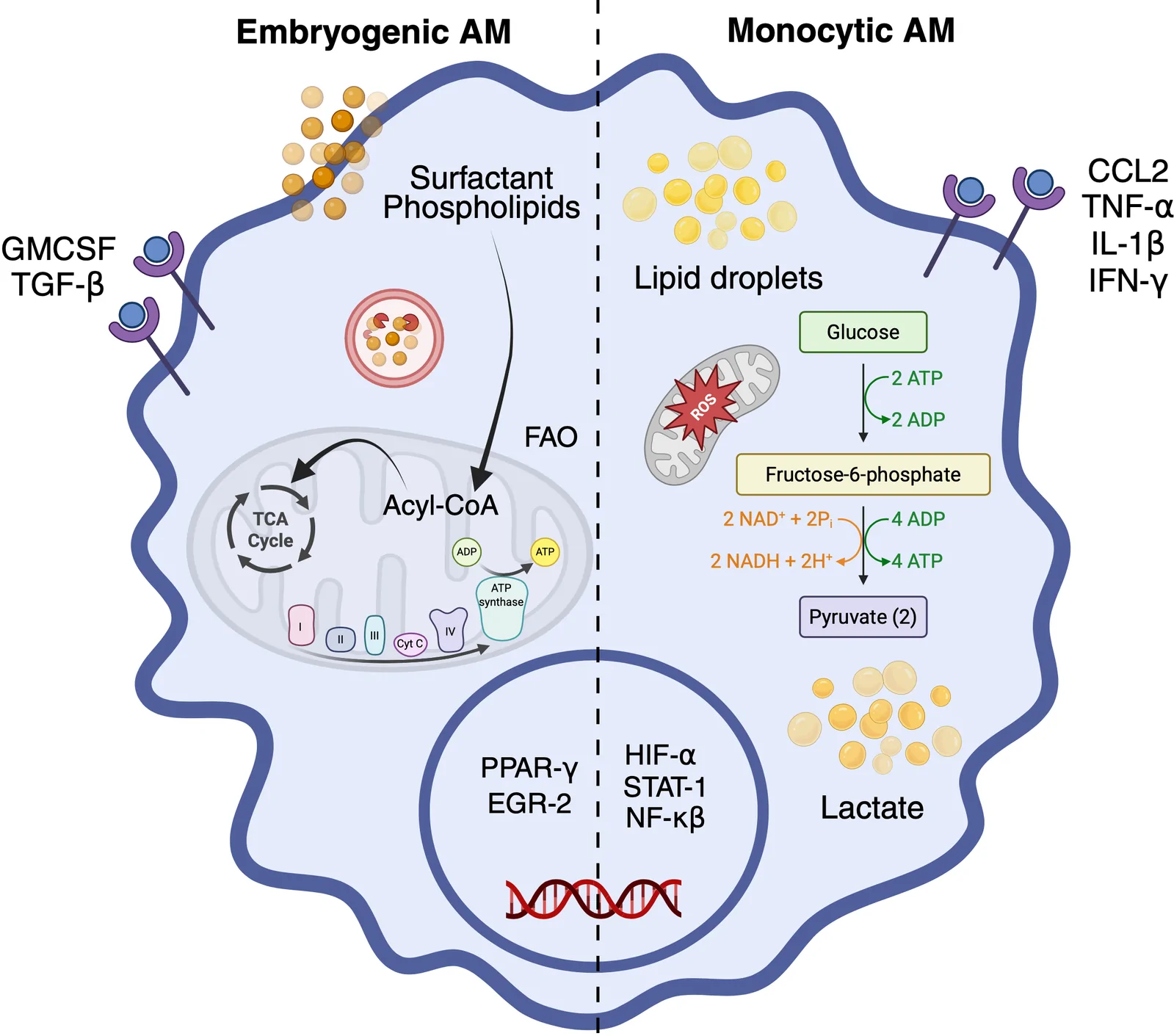
Homeostatic, embryonic AMs and inflammatory, monocytic AMs display distinct metabolic and transcriptional profiles. This image depicts differences between embryonic AMs under steady-state conditions (left) with recruited monocyte-derived AMs during inflammation (right). Distinct environmental signals shape the metabolic and functional states of embryonic and monocytic AMs. GM-CSF and TGF-β support embryonic AM differentiation and maintenance, whereas CCL2, TNF-α, and IL-1β promote differentiation and inflammatory remodeling of monocytic AMs. Unlike resident AMs at homeostasis, monocytic AMs exhibit increased glycolytic metabolism, lactate production, and lipid droplet accumulation to drive pro-inflammatory effector functions.

Upon activation, AM metabolism can be reprogrammed to promote antimicrobial activity. However, AM programming differs from classical macrophage activation paradigms described in bone marrow-derived macrophages. In many macrophage types, exposure to microbial ligands and inflammatory cytokines activates signaling such as HIF-1α and mTOR, increasing glycolytic enzyme expression and glucose use for inflammatory insults (Tannahill et al., 2013; Kelly and O'Neill, 2015). In contrast, murine and human AMs blunt glycolytic activity in responses to lipopolysaccharide (LPS) and can maintain OXPHOS for cytokine production, whereas monocyte-derived macrophages increase glycolytic flux and lactate production (Woods et al., 2020; Pereverzeva et al., 2022). However, human airway macrophages can engage glycolysis under certain inflammatory conditions, suggesting contact dependent metabolic responses (Cox et al., 2024). Under pathological hypoxia conditions in alveoli beyond injury or inflammation, HIF-1α stabilization alters metabolic signatures in tissue-resident AMs, increasing glycolytic markers to adapt to low oxygen levels rather than triggering a canonical inflammatory glycolytic switch (Woods et al., 2022). AM activation is less dependent on classical HIF-1α-driven glycolytic reprogramming than other macrophage populations. Ultimately, activated AM metabolic reprogramming is context-specific and shaped by origin and pulmonary and alveolar signaling cues. Consequently, AM activation differs from the classical glycolysis-centric macrophage model (**Figure 2**).

## Bacterial reprogramming of alveolar macrophage metabolism

As sentinels of the lung, AMs continuously phagocytose inhaled microbes and debris to maintain pulmonary homeostasis. However, intracellular bacteria defend themselves by exploiting key cellular processes to protect a pro-bacterial growth niche. As discussed above, macrophage metabolism is a central process linked to cell survival and immune responses, making it an attractive target for pathogens. Below, we discuss examples of four intracellular bacteria that establish a pulmonary niche by manipulating AM metabolism (**Table 1**).

**Table 1:** AM metabolic pathways manipulated by pulmonary intracellular bacterial pathogens.

Pathogen	Intracellular metabolic strategy	Metabolic pathways manipulated
*Mycobacterium tuberculosis*	Exploits oxidative and lipid-rich AM metabolism	FAO, lipid and cholesterol metabolism, OXPHOS
*Coxiella burnetii*	Preserves mitochondrial and lipid homeostasis	OXPHOS, mitochondrial fragmentation, cholesterol trafficking, lipid metabolism
*Legionella pneumophila*	Rewires metabolism to aerobic glycolysis	Mitochondrial fragmentation, glycolysis, lactate production, ER-autophagy interactions
*Francisella tularensis*	Suppresses inflammatory metabolic activation	Glycolytic suppression, OXPHOS, lactate oxidation, TCA cycle

## Mycobacterium tuberculosis

*Mycobacterium tuberculosis* is a facultative intracellular bacterium, a global pathogen that causes acute and persistent respiratory tuberculosis following infection of AMs, wherein the bacterium generates an intracellular niche reminiscent of early to mid-phagosomes that contains few proteases and low acidity (Cohen et al., 2018; Huang et al., 2018). Early stages of tuberculosis occur within AMs, and *M. tuberculosis* exploits host metabolism to facilitate bacterial growth. Unlike many macrophage types that undergo glycolytic reprogramming upon *M. tuberculosis* infection, AMs have a dampened glycolytic response and retain an oxidative metabolic state associated with bacterial permissiveness. AMs are intrinsically lipid-driven and oxidative, relying on FAO and OXPHOS, and macrophage origin and metabolic state dictate bacterial permissiveness. As a prime example, AMs support *M. tuberculosis* replication, while recruited interstitial macrophages (IMs) exhibit glycolytic metabolism and restrict bacterial growth (Huang et al., 2018). Both murine and human AMs exhibit impaired transition from OXPHOS to glycolysis after *M. tuberculosis* infection and are unable to mount metabolic responses required for antimicrobial activity (Huang et al., 2018; Mendonca et al., 2022). In contrast to bone marrow-derived macrophages, where a glycolytic shift supports bacterial control, AMs maintain an oxidative state that correlates with increased microbial replication (Huang et al., 2018). Pharmacologic reprogramming of AMs to glycolysis represses bacterial growth, indicating that metabolism underlies macrophage permissiveness for bacterial *M. tuberculosis* replication (Mendonca et al., 2022). Macrophage metabolism and nutrient availability are predicted to control the divergent *M. tuberculosis* fate in AMs and IMs. Supporting this hypothesis, *M. tuberculosis* uses host-derived fatty acids and cholesterol as carbon sources during intracellular infection. AMs are enriched in lipid and cholesterol processing pathways that provide a metabolically favorable niche for bacterial persistence. Disruption of host cholesterol trafficking or bacterial cholesterol catabolism impairs intracellular survival, highlighting the dependence of *M. tuberculosis* on host-derived sterols during chronic infection (Pandey and Sassetti, 2008; Miner et al., 2009; Kim et al., 2010). Collectively, these findings demonstrate that macrophage metabolism is exploited by *M. tuberculosis* to program AMs as a pro-bacterial niche driven by fatty acid oxidation and OXPHOS.

The inability of AMs to mount glycolysis-driven antimicrobial protection against *M. tuberculosis* is reflected at the transcriptional level in human disease. AMs isolated from tuberculosis patients have altered inflammatory gene expression, with dysregulated cytokine signaling, impaired phagocyte receptor expression, and altered lipid-associated pathways that are critical for AM viability (Lavalett et al., 2017). These changes reflect a state of incomplete AM activation, where a lack of inflammatory signaling may contribute to poor bacterial control. In addition, human AMs produce significantly less itaconate than monocyte-derived macrophages, and impaired itaconate formation increases permissiveness to *M. tuberculosis* (Krebs et al., 2026). Itaconate is generated by the TCA cycle intermediate cis-aconitate by IRG1/ACOD1 and serves as an AM antimicrobial metabolite that inhibits bacterial isocitrate lyase, disrupting *M. tuberculosis* metabolism in a detrimental manner (Michelucci et al., 2013). Beyond its direct antimicrobial activity, itaconate activates Nrf2 signaling through KEAP1 alkylation, promoting nuclear translocation of Nrf2 and induction of antioxidant, stress-response, and immunoregulatory genes. This positions itaconate as both an antimicrobial metabolite and a regulator of macrophage inflammatory responses (Mills et al., 2018). *M. tuberculosis* reliance on altered metabolite abundance suggests that metabolic kinetics are macrophage subtype-specific and impact distinct antimicrobial events.

Following *M. tuberculosis* infection, human AMs produce nitric oxide (NO), and higher NO levels correlate with reduced intracellular bacterial growth (Rich et al., 1997). However, this response is variable and insufficient, reflecting a partial AM antimicrobial response characterized by impaired glycolytic programming, reduced itaconate production, and sustained OXPHOS, all of which restrict full antimicrobial function (Mendonca et al., 2022; Krebs et al., 2026). To counter oxidative insults, *M. tuberculosis* engages antioxidant programs, including Nrf2 activation mediated by ESX-1 secreted ESAT-6, that limit oxidative stress and suppress antimicrobial functions (Rothchild et al., 2019). Mechanistically, Nrf2 induces cytoprotective and antioxidant gene expression, reduces reactive oxygen species production, and suppresses pro-inflammatory signaling, ultimately preserving mitochondrial function. These Nrf2-regulated activities collectively generate an oxidative and immunosuppressive niche that supports bacterial persistence (Rothchild et al., 2019). Taken together, the findings above indicate that *M. tuberculosis* exploits AM intrinsic metabolic activity rather than extensively reprogramming metabolism. By maintaining an oxidative, lipid-dependent state, limiting glycolytic activation, and engaging antioxidant responses, *M. tuberculosis* establishes a permissive intracellular niche within AMs for prolonged replication.

## Coxiella burnetii

*Coxiella burnetii* is a Gram-negative obligate intracellular pathogen that causes Q fever and establishes a primary replicative niche within AMs following inhalation by a host. Upon encountering an AM, *C. burnetii* facilitates uptake into an early endosome that continuously fuses with autophagosomes and lysosomes to form the *C. burnetii* growth compartment. Fusion with lysosomes metabolically activates *C. burnetii* to produce a type IV secretion system (T4SS) and deploy ~120 effectors that manipulate host signaling pathways and engage organelles to benefit the pathogen. This specialized lysosome-like vacuole, termed the *Coxiella*-containing vacuole (CCV), is essential for bacterial growth and disease progression, and the lung represents the initial site of infection (Voth and Heinzen, 2007; Dragan et al., 2019).

*C. burnetii* manipulates several host functions in a T4SS-dependent manner to orchestrate a productive infection cycle, including vesicular trafficking, autophagosome fusion, inhibition of host cell death, and engagement of host cell signaling, including the unfolded protein response and NRF2 pathway (Voth et al., 2007; Beare et al., 2011; Winchell et al., 2014; Winchell et al., 2018; Brann et al., 2020). Activation of the UPR and Nrf2 signaling helps *C. burnetii* adapt to macrophage antimicrobial responses by limiting oxidative stress and preserving mitochondrial homeostasis, while inhibition of apoptosis maintains a sustained intracellular niche required for bacterial replication. Mitochondria, the powerhouses of AMs and organelle supporting OXPHOS, are targeted by *C. burnetii* for metabolic and survival purposes. Multiple secreted *C. burnetii* effector proteins localize to mitochondria, where they alter mitochondrial morphology, membrane potential, physiology, and apoptotic signaling without inducing overt organelle damage (Fielden et al., 2017; Fielden et al., 2021; Yek et al., 2023; Adhvaryu et al., 2025). A class of novel proteins that were identified using proteomic approaches, known as mitochondrial *Coxiella* effector proteins (Mces), localize to mitochondria and interact with organelle quality control machinery (Fielden et al., 2021). C*. burnetii* preserves mitochondrial function and sustains OXPHOS, aligning with baseline AM metabolism. Furthermore, mitochondrial modulation enhances AM OXPHOS and prevents apoptosis to support bacterial persistence (Loterio et al., 2023; Adhvaryu et al., 2025).

In addition to directly engaging mitochondria, *C. burnetii* growth is restricted by itaconate and HIF-1α activity. Induction of itaconate via IRG1 signaling and HIF-1α stabilization are associated with inflammatory metabolic pathways in AMs. HIF-1α activates inflammatory responses fueled by glycolysis that are detrimental to *C. burnetii*, specifically by limiting intracellular availability of citrate, an essential nutrient required for bacterial replication. Meanwhile, itaconate further limits replication by disrupting *C. burnetii* metabolism, linking host metabolism to bacterial control (Hayek et al., 2022; Kohl et al., 2023). These findings suggest *C. burnetii* adapts to a narrow metabolic window, preferring OXPHOS over excessive glycolytic metabolism. Therefore, maintaining homeostatic OXPHOS metabolism in AMs and minimizing metabolic disruption allows the pathogen to sustain a permissive growth niche.

AMs also depend on lipid metabolism, relying on fatty acid oxidation, continuous cholesterol trafficking, and surfactant processing to sustain homeostasis (Baker et al., 2010; Schneider et al., 2014). Lipid dependence creates a metabolic opportunity for *C. burnetii*, and the pathogen exploits host lipid metabolism, particularly cholesterol trafficking, to support CCV expansion and bacterial replication. Cholesterol dynamics within the host cell and CCV are important determinants of *C. burnetii* infection. While cholesterol contributes to bacterial uptake and CCV biogenesis, excessive cholesterol accumulation within the CCV promotes acidification and bacterial death, highlighting the importance of regulated sterol trafficking during infection. Consistent with this finding, pharmacologic disruption of cholesterol transport or biosynthesis impairs vacuole development and limits bacterial growth, indicating *C. burnetii* depends on controlled cholesterol homeostasis rather than simple cholesterol abundance (Howe and Heinzen, 2006; Gilk et al., 2013; Mulye et al., 2017). Currently, regulation of lipid metabolism and cholesterol dynamics in surfactant-conditioned, *C. burnetii*-infected human AMs remains undefined. Thus, further investigation of the interplay between mitochondrial regulation and lipid metabolism should uncover a coordinated strategy in which metabolism, membrane dynamics, and CCV expansion are tightly linked. Collectively, current research indicates that *C. burnetii* hijacks existing AM metabolic pathways to promote persistence within the host. This conclusion is evidenced by *C. burnetii* establishment of an intracellular growth niche while preserving mitochondrial function, limiting inflammatory metabolic pathways, and modulating intracellular cholesterol. However, many mitochondrial and cholesterol trafficking events described above have been characterized in macrophage model systems, and their contribution to *C. burnetii* infection in primary human AMs is not fully defined.

## Legionella pneumophila

*Legionella pneumophila* is a Gram-negative facultative intracellular pathogen that causes pulmonary Legionnaires’ disease and establishes a phagosomal replicative niche within macrophages following inhalation. Unlike *C. burnetii*, *L. pneumophila* actively avoids lysosomal fusion following phagocytosis, remaining within a specialized membrane-bound compartment termed the *Legionella*-containing vacuole (LCV). Using a T4SS, *L. pneumophila* facilitates continual LCV fusion with host endoplasmic reticulum (ER) membrane and autophagosome components, while reprogramming host processes to support bacterial survival and replication. Cellular remodeling required for LCV formation promotes a biphasic life cycle characterized by an initial intracellular replicative phase that switches to a flagellated, virulent transmissive phase upon nutrient exhaustion (Isberg et al., 2009; Qiu and Luo, 2017). However, in contrast to the relatively oxidative and quiescent metabolic strategy of *C. burnetii*, *L. pneumophila* actively drives host metabolic reprogramming toward a glycolytic, inflammatory state. *L. pneumophila* triggers mitochondrial fragmentation through a T4SS-dependent mechanism involving the effector MitF, which promotes Drp1-mediated mitochondrial fission and disrupts organelle network integrity (Escoll et al., 2017). Concurrently, the T4SS effector LamA degrades host glycogen to substantially increase cytosolic glucose pools (Price et al., 2020). This remodeling is associated with reduced mitochondrial respiration and a compensatory increase in glycolytic flux, characterized by increased glucose uptake and lactate production. Importantly, inhibiting mitochondrial fission or glycolysis restricts bacterial replication, demonstrating that these metabolic changes are functionally linked to intracellular growth rather than secondary consequences of infection (Escoll et al., 2017). Although these studies were primarily performed in bone marrow-derived macrophages and human cell lines, they provide a mechanistic framework for how *L. pneumophila* overrides the oxidative metabolic program of AMs.

Collectively, *L. pneumophila* exemplifies a metabolic strategy distinct from other intracellular pulmonary pathogens. Rather than exploiting native macrophage oxidative metabolism, the pathogen actively triggers mitochondrial fragmentation and glycolytic reprogramming to support replication and drive host inflammation. This contrast demonstrates that intracellular pathogens differentially exploit and reshape macrophage metabolism, with ultimate infection outcomes determined by alteration of the metabolic identity of the infected tissue.

## Francisella tularensis

*Francisella tularensis* is a facultative intracellular pathogen that establishes a primary niche within AMs and causes tularemia, a disease characterized by severe pneumonia, fever, chest pain, and lymphadenopathy (Sjostedt, 2007; Roberts et al., 2014). Although *F. tularensis* can infect other cell types, including alveolar epithelial cells, early replication and immune evasion are primarily defined within AMs (Roberts et al., 2014). Upon entry into AMs, *F. tularensis* resides within a *Francisella*-containing phagosome (FCP). The FCP matures through the endocytic pathway, after which *F. tularensis* escapes into the cytosol. Metabolic strategies are manipulated during early stages of infection, with NADPH oxidase driving an initial antimicrobial oxidative burst within the FCP that *F. tularensis* counters by using host glutamate to fuel the bacterial TCA cycle and mount antioxidant defenses. *F. tularensis* subsequently replicates within the cytosol while sequestering host cell metabolites and nutrients. Following replication, *F. tularensis* relies on host cell death driven by mitochondrial depolarization for dissemination and cell-to-cell spread (Ramond et al., 2014; Wyatt et al., 2016; Degabriel et al., 2023).

*F. tularensis* manipulates macrophage metabolism to orchestrate a pro-bacterial intracellular growth environment. *F. tularensis* maintains macrophages in a predominantly OXPHOS- and FAO-driven metabolic state (Wyatt et al., 2016; Jessop et al., 2025). During initial stages of infection, macrophages fail to mount sufficient inflammatory responses, suggesting the pathogen activates immunosuppressive signaling pathways. *F. tularensis* prevents the shift from OXPHOS to aerobic glycolysis, thereby suppressing pro-inflammatory cytokine production and antimicrobial functions. Virulent strains of *F. tularensis* suppress HIF-1α stabilization and fail to induce key glycolytic regulators, effectively blocking metabolic reprogramming required for inflammatory activation (Wyatt et al., 2016).

*F. tularensis* also regulates mitochondrial function in parallel with the metabolic changes above. During early stages of infection, *F. tularensis* sustains and enhances mitochondrial respiration, preserving OXPHOS and preventing host cell death. These activities stabilize the intracellular growth niche while limiting inflammatory signaling required for bacterial clearance. Temporal regulation of mitochondrial activity reflects a strategy by which organelle function is maintained only as long as necessary to support replication, followed by mitochondrial depolarization and host cell death during later stages of infection to facilitate dissemination (Jessop et al., 2018).

Beyond suppressing glycolysis, *F. tularensis* selectively exploits host carbon metabolism. Through its O-antigen, the bacterium promotes lactate uptake and oxidation, fueling the TCA cycle and sustaining mitochondrial ATP production (Jessop et al., 2025). This metabolic routing supports redox balance, limits inflammatory signaling, and preserves host cell viability. In parallel, bacterial lipids engage receptors such as TLR2 and PPAR-α to further suppress cytokine production, reinforcing a metabolically restrained state (Crane et al., 2013). Within AMs, this strategy aligns with the baseline oxidative and low inflammatory environment of the lung. Initially, *F. tularensis* prevents the metabolic shift required for macrophage activation and antimicrobial activity, providing time for bacterial replication. By sustaining OXPHOS, blocking glycolysis, and selectively exploiting host-derived metabolites, *F. tularensis* maintains a permissive pro-bacterial intracellular compartment. Most findings above have been reported in bone marrow-derived macrophages, which have metabolic profiles distinct from AMs. Nonetheless, current evidence suggests a dynamic relationship between *F. tularensis*, host metabolism, and mitochondrial processes.

## Discussion

Successful intracellular pathogen infection depends on the ability of a bacterium to establish a replicative niche within a host cell. Unlike extracellular pathogens, these organisms must preserve host cell viability long enough to sustain nutrient acquisition, replication, and dissemination while simultaneously suppressing antimicrobial responses. Hence, host metabolism is an attractive and perhaps essential target for intracellular pathogens. Within the lung, AMs present a uniquely permissive cellular environment shaped by active mitochondrial respiration, fatty acid oxidation, surfactant metabolism, and restrained inflammatory signaling required to preserve pulmonary homeostasis. Metabolic programs that maintain gas exchange and prevent excessive inflammation can be used by intracellular pathogens. Among the pathogens discussed herein, intracellular persistence relies on manipulation of mitochondria, lipid metabolism, and inflammatory signaling subversion (Hussell and Bell, 2014; Andrews et al., 2022). For example, *M. tuberculosis* predominantly exploits AM OXPHOS and FAO, *C. burnetii* preserves mitochondrial and lipid homeostasis, *L. pneumophila* actively rewires mitochondrial metabolism toward glycolysis and inflammation, and *F. tularensis* suppresses glycolytic activation while temporally regulating mitochondrial activity. AMs provide a permissive niche to these pathogens that have a repertoire of proteins that hijack AM processes while adapting to metabolism at homeostasis. AM metabolism is ultimately equipped to avoid excessive inflammation, providing an opportunity for these stealth pathogens to hide within the enemy lines and avoid immune detection.

*F. tularensis* and *L. pneumophila* exhibit shorter intracellular lifecycles compared to *M. tuberculosis* and *C. burnetii*. Following uptake by AMs, *F. tularensis* and *L. pneumophila* complete intracellular growth and egress within 24–48 h (Isberg et al., 2009; Wyatt et al., 2016), while *M. tuberculosis* and *C. burnetii* persist for several days to weeks (Voth and Heinzen, 2007; Cambier et al., 2014). These differences may reflect the distinct metabolic strategies used to support intracellular survival. *F. tularensis* and *L. pneumophila* initially manipulate mitochondrial metabolism to promote replication, but later transition toward mitochondrial dysfunction and host cell death coinciding with bacterial egress. In contrast, the lengthy *M. tuberculosis* and *C. burnetii* replication cycles align with intrinsic AM metabolism, preserving OXPHOS, FAO, mitochondrial integrity, and lipid metabolism while maintaining host cell viability over prolonged periods. Despite these differences, the pathogens discussed here ultimately rely on sequestration of host-derived metabolites to sustain intracellular growth. For example, *M. tuberculosis* uses host fatty acids and cholesterol as major carbon sources, while *C. burnetii* depends on citrate availability and tightly regulates cholesterol trafficking to the CCV, and *F. tularensis* redirects lactate and TCA cycle metabolism to support mitochondrial ATP production. Simultaneously, several pathogens actively evade antimicrobial metabolites, including itaconate and HIF-1α–associated inflammatory signaling, which restrict intracellular replication by limiting bacterial access to essential metabolites or directly disrupting bacterial metabolism (Wyatt et al., 2016; Roberts et al., 2022). In contrast, the pulmonary extracellular pathogens *Yersinia pestis* and *Acinetobacter baumannii* largely avoid the AM intracellular environment. Instead of preserving macrophage viability and manipulating intracellular metabolism, these pathogens primarily evade phagocytosis, resist extracellular immune defenses, acquire nutrients from damaged tissue environments, and trigger inflammatory pathology that promotes bacterial dissemination. *Y. pestis* transiently survives within macrophages during early stages of infection but rapidly transitions to extracellular growth accompanied by potent suppression of phagocytosis and inflammatory signaling (Pujol and Bliska, 2005). Similarly, *A. baumannii* predominantly persists extracellularly within inflamed pulmonary environments, relying on biofilm formation, oxidative stress resistance, iron acquisition, and metabolic adaptation to a nutrient-limited region (Mortensen and Skaar, 2013). Collectively, these comparisons demonstrate that intracellular pathogens uniquely exploit host metabolism to facilitate persistence.

The pulmonary environment is a specialized metabolic landscape characterized as oxygen-rich, with continuous surfactant turnover, abundant lipid availability, and tightly regulated immune signaling. These features lead to a unique AM metabolic signature enriched for mitochondrial respiration, OXPHOS, FAO, cholesterol trafficking, and surfactant catabolism that differs substantially from peripheral macrophage populations (Hussell and Bell, 2014). Many studies highlighted in this review relied on bone marrow-derived macrophage models that have fundamentally distinct metabolic and inflammatory responses from resident AMs. This difference is important because many intracellular respiratory pathogens, including *M. tuberculosis*, *C. burnetii*, *L. pneumophila*, and *F. tularensis*, establish their primary replicative niche within AMs *in vivo*. Consequently, macrophage models lacking the metabolic, developmental, and environmental features of AMs may incompletely capture all human disease-relevant events. This review highlights a gap in the field of pulmonary immunometabolism and the need for models that capture the unique metabolic identity of AMs. Major knowledge gaps remain regarding mechanisms by which intracellular pathogens reprogram primary human AM metabolism, how findings from BMDMs translate to AMs, and how pulmonary environment signals shape host-pathogen metabolic-interactions. Investigating these questions will identify metabolism-based therapeutic strategies to combat infections. Greater use of primary human AMs will likely provide systems that most accurately reflect human pulmonary disease. However, widespread use of human AMs remains technically challenging due to limitations in accessibility, isolation procedures, donor variability, and long-term maintenance *ex vivo*. Additionally, challenges arise from the lack of current models that fully capture the pulmonary microenvironment, including epithelial crosstalk, oxygen gradients, surfactant exposure, and tissue-specific signaling during infection (Liu et al., 2025). Increasing evidence also suggests that AMs do not represent a metabolically uniform population. Recent studies reveal substantial diversity in AM origins, activation states, and metabolic programs, moving the field away from a singular view of AM identity (Mould et al., 2019). Instead, AMs appear to exist along a spectrum of metabolic and inflammatory states capable of mounting distinct immune responses. Investigating whether intracellular pathogens preferentially target specific metabolically-permissive AM subsets during infection will broaden our understanding of host-pathogen interactions within the lung and reveal additional determinants of bacterial persistence.

Expanding our understanding of AM metabolism and bacterial reliance on specific metabolic components will guide future therapeutic interventions and vaccine development aimed at selectively altering metabolic dynamics to enhance pulmonary immunity while preserving lung homeostasis. Collectively, current research positions AM metabolism at the intersection of breathing and burning, where metabolism that preserves pulmonary homeostasis simultaneously shapes inflammatory responses, ultimately controlling the fate of intracellular bacterial pathogens.
